# Appropriateness of transferring nursing home residents to emergency departments: a systematic review

**DOI:** 10.1186/s12877-019-1028-z

**Published:** 2019-01-21

**Authors:** Sabine E Lemoyne, Hanne H. Herbots, Dennis De Blick, Roy Remmen, Koenraad G. Monsieurs, Peter Van Bogaert

**Affiliations:** 1 Emergency Department, Antwerp University Hospital, Wilrijkstraat 10, 2650 Edegem, Belgium; 2 Faculty of Medicine and Health Sciences, University of Antwerp, Universiteitsplein 1, 2610 Wilrijk, Belgium; 3 Department of Primary and Interdisciplinary Care, Universiteitsplein 1, 2610 Wilrijk, Belgium; 4 Center for Research and Innovation in Care, Universiteitsplein 1, 2610 Wilrijk, Belgium

**Keywords:** Appropriateness, Emergency medical service, Emergency department, General practitioner, Nursing home, Nursing staff, Patient transfer

## Abstract

**Background:**

Elderly living in a Nursing Home (NH) are frequently transferred to an Emergency Department when they need acute medical care. A proportion of these transfers may be considered inappropriate and may be avoidable.

**Methods:**

Systematic review. Literature search performed in September 2018 using PubMed, Web of Science, the Cochrane Library and the Cumulative Index to Nursing and Allied Health Literature database. Titles and abstracts were screened against inclusion and exclusion criteria. Full-texts of the selected abstracts were read and checked for relevance. All years and all languages were included provided there was an English, French, Dutch or German abstract.

**Results:**

Seventy-seven articles were included in the systematic review: 1 randomised control trial (RCT), 6 narrative reviews, 9 systematic reviews, 7 experimental studies, 10 qualitative studies and 44 observational studies. Of all acute transfers of NH residents to an ED, 4 to 55% were classified as inappropriate. The most common reasons for transfer were trauma after falling, altered mental status and infection. Transfers were associated with a high risk of complications and mortality, especially during out-of-hours. Advance directives (ADs) were usually not available and relatives often urge NH staff to transfer patients to an ED. The lack of availability of GPs was a barrier to organise acute care in the NH in order to prevent admission to the hospital.

**Conclusions:**

The definition of appropriateness is not uniform across studies and needs further investigation. To avoid inappropriate transfer to EDs, we recommend to respect the patient’s autonomy, to provide sufficient nursing staff and to invest in their education, to increase the role of GPs in the care of NH residents both in standard and in acute situations, and to promote interprofessional communication and collaboration between GPs, NH staff and EDs.

**Electronic supplementary material:**

The online version of this article (10.1186/s12877-019-1028-z) contains supplementary material, which is available to authorized users.

## Background

In 2017 the percentage of elderly people (65 years and older) in Europe accounted for 19.4% of the total population. Over the last 10 years this proportion has increased by 2.4% and is expected to increase further to 24% in 2030 [1]. Ageing will also increase the demand for more acute medical care. Many nursing home (NH) residents are frail with multiple chronic conditions [2–17]. When NH residents have an acute exacerbation or a complication of their chronic illness, an injury (fall-related) or acute infection, they may require acute medical services [11, 12, 14, 18]. General practitioners (GPs), nurses, emergency medical services and emergency departments (EDs) play central roles in providing acute medical care in these situations.

There is a thin line between appropriate and inappropriate ED transfers of older people living in NHs [14, 15, 19, 20]. Studies often raise awareness about the striking numbers of inappropriate ED transfers of NH residents. They report various reasons for these ED transfers and suggest several solutions. Some authors have attempted to define appropriateness of ED transfers. This is a difficult task because inappropriateness remains largely subjective. The purpose of this systematic review is to define the characteristics of ED transfers of NH residents, to describe definitions of appropriateness and to identify factors associated with a reduction in inappropriate transfers.

## Methods

### Search strategy and inclusion criteria

A literature search was performed on September 2018 using PubMed, Web of Science, the Cochrane Library and the Cumulative Index to Nursing and Allied Health Literature (CINAHL) database. The following search was performed in Pubmed using MeSH terms: [“Nursing Homes” OR “Homes for the Aged” OR “Housing for the Elderly” OR “Residential Facilities” OR “Long-Term Care”] AND “Emergency Medical Services”. Because there was no available MeSH term for “appropriateness”, the search was repeated using keywords: “Nursing Homes” OR “Homes for the Aged” OR “Housing for the Elderly” OR “Residential Facilities” OR “Long-Term Care” AND “Emergency Medical Services” AND “Appropriateness”. Subsequent searches were performed in Web of Science, the Cochrane library and CINAHL.

Titles and abstracts were assessed against inclusion and exclusion criteria, full-text articles were reviewed when relevance was established. The inclusion criteria were: patients of 65 years and older, living in a NH where nursing staff are responsible for the care of the residents. For the purpose of this review the term NHs also included residential aged care facilities, care homes, continuing care retirement community sites, extended care facilities, long-term care facilities and skilled nursing facilities. The included studies contained information on the ED transfer or on the primary care provided at the NHs. Our primary focus was to find information concerning the appropriateness of ED transfers. Studies about finance and transfer forms were excluded. All years and all languages were included provided there was an English, French, Dutch or German abstract.

### Study selection and data extraction

Two reviewers (HH and DDB) independently screened titles and abstracts, selecting those meeting the inclusion criteria. In case of disagreement or uncertainty, a third reviewer (SL) took the final decision. The full-text articles were then reviewed and articles of no or low relevance were excluded. Two reviewers (HH and DDB) extracted the data. Systematic reviews were assessed by the AMSTAR checklist [21] and narrative reviews were assessed by the JBI Critical Appraisal Checklist for Narrative Expert opinion & text [22]. Experimental studies were assessed by the CONSORT 2010 checklist for RCTs [23] and by the JBI Critical Appraisal Checklist for Quasi-Experimental Studies [24]. The STROBE [25] and COREQ [26] checklists were used to rate observational studies and qualitative studies. (see Additional file 1, Appendix 1–6) No articles were excluded based on their quality.

## Results

### Search results

**Fig. 1:**
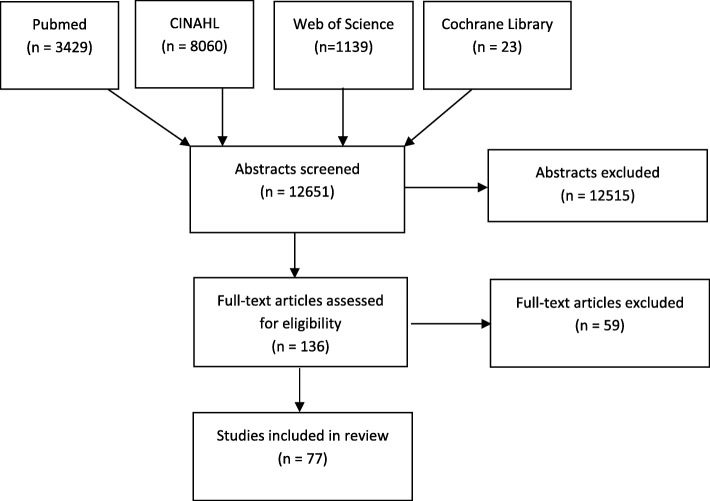
Prisma 2009 Flow Diagram

The Pubmed search identified 3429 articles. Searching Web of Science, the Cochrane Library and the CINAHL database identified 1139, 23 and 8060 articles, respectively. Duplicate articles were excluded. Twelve thousand six hundred and fifty-one titles and abstracts were reviewed against the inclusion criteria and for relevance. Twelve thousand five hundred and fifteen papers were excluded because of lack of relevance. After full-text review of the remaining 136 papers, 77 were selected for data extraction. (see Fig. 1).Of the 77 included studies 71% were published between 2011 and 2018, most were from Europe (25 studies) and from the United States (22 studies) (Table 1). There was 1 randomised control trial (RCT), 6 narrative reviews, 9 systematic reviews, 7 experimental studies, 10 qualitative studies and 44 observational studies. The quality assessment of the articles is presented in Appendix 1–6 (see Additional file 1).

**Table 1:** Distribution of reviewed studies by country and year of publication intervals

Country	Number of studies			
	1990–1999	2000–2005	2006–2010	2011–2018
United Kingdom	0	1	3	8
Australia	0	0	5	13
Ireland	0	0	0	2
United States	1	4	1	16
Canada	0	1	3	6
Sweden	0	0	0	1
Switzerland	0	0	0	2
Singapore	0	1	0	0
Norway	0	0	1	1
Austria	0	0	0	1
France	0	0	0	2
Germany	0	0	0	1
Portugal	0	0	0	1
Belgium	0	0	1	1

### Characteristics/outcomes of all transfers

Across all studies, the most common reasons for ED transfer from the NH were trauma, altered mental state and infection (Table 2). Gruneir et al. found that 87% of all ED admissions of NH residents were fall-related [27]. Potentially preventable (and thus judged inappropriate) ED visits were injuries related to falling, fever, decreased food or fluid intake, functional decline, shortness of breath, new urinary incontinence, heart conditions and pneumonia [27–32].

**Table 2:** Common reasons for ED transfer

Common reasons for ED transfers		Articles
Trauma	2–59%	[2–6, 16, 19, 27, 28, 35, 37, 39, 40, 45, 49, 68, 70, 71]
Altered mental state	1–41%	[3, 5, 6, 27, 37, 39, 71]
Infection/inflammation	3–49%	[3, 19, 27, 35, 40]
Respiratory	9–30%	[2–6, 8, 16, 18, 19, 27, 28, 37, 45, 49, 70]
Genitourinary	5–23%	[2, 3, 5, 6, 8, 16, 18, 19, 27, 28]
Gastrointestinal	6–22%	[2, 5, 6, 8, 19, 27, 37, 39, 45, 49, 70]
Catheter problems	4–15%	[37, 39]
Cardiovascular	3–21%	[2, 4, 6, 8, 16, 19, 27, 45, 49, 70]
Fever	5–13%	[6, 37]
Central Nervous System symptoms	3–9%	[4, 8, 19, 27, 39, 49]

Of all NH residents who were transferred to an ED, 1 to 2% died during transfer or in the ED. [3, 6, 7, 10, 30, 33, 34] Only 23 to 53% of the NH residents visiting the ED were discharged the same day [3, 5, 7, 28, 34, 35]. NH residents who were admitted to the hospital showed a high risk for delirium [2, 36], falls [2, 31], hospital acquired infections [2], iatrogenic complications [2, 31, 36–38], and functional decline [2, 31, 37]. The inpatient mortality varied from 6 to 25% [3, 7, 10, 16, 30, 33, 39] and the 1 month mortality ranged from 4 to 24% [27, 33]. Carron et al. reported an overall mortality of 14% [4]. When considering only the inappropriate transfers, 62% were admitted in the hospital and 24% died within 30 days [40].

### .Appropriateness of transfer metrics

Across all studies 4 to 55% of ED transfers were classified as inappropriate [2, 3, 5, 18, 27, 29, 30, 32, 35, 39–46]. The definition of inappropriateness varied widely between studies (see Additional file 2: Table S3). Some authors defined inappropriate ED transfers as preventable transfers; a transfer that may have been avoided if an existing condition would have been managed optimally in the NH at an earlier stage or when adequate prevention would have avoided its initial presentation [2, 27, 29, 45, 47]. Other authors defined inappropriateness by using a list of symptoms and conditions that were frequently associated with potentially preventable ED visits and hospitalisations [18, 27, 29, 31, 33, 44–46, 48]. On the other hand, some authors defined appropriate ED admissions using a list of symptoms and conditions certainly needing acute medical attention [5, 18, 45, 46]. Jensen et al. suggested that appropriateness of referral should be defined as a balance between timeliness, availability of diagnostic and treatment resources (e.g. intravenous access, oxygen, drugs), timely test results, physician and nursing availability and expertise, advance directives, respect for patient or family wishes, availability of background medical information, and comorbidity [49]. Saliba et al. defined appropriateness as the lowest level of safe care for a patient with a specific presentation [41]. Thus, inappropriate transfers represent situations in which care in a lower cost setting (i.e. the NH) would be as safe as and less disruptive than care in a higher cost hospital setting. They identified several factors influencing a physician’s choice to judge a transfer as inappropriate: the patient’s chief complaint did not mandate hospitalisation, the patient’s acute condition did not increase the probability of death or decline in functional status, the patient’s acute condition did not require immediate evaluation, management at the NH had not been tried or the required services are available on an outpatient basis. A survey among NH staff revealed that inadequate end-of-life care planning, inadequate education of NH staff, unavailability of physicians and pressure exerted on NH staff by families were common reasons for inappropriate transfer to the ED [50].

### Factors that influence the proportion of appropriate transfers

ED transfers of NH residents increased the risk of complications and mortality especially during out-of-hours [2, 5, 9, 11, 12, 15, 17, 20, 30, 51]. Reducing inappropriate ED transfers may lower health care costs, reduce harm and complications resulting from medical treatment and improve quality of care [29, 35, 45].

Advance directives help physicians and NH staff to make appropriate decisions, thus decreasing inappropriate ED transfers, unwanted resuscitation and hospital admissions [10, 36, 41, 49, 51–55]. Studies show that NH residents hope that they will not be hospitalised, particularly at the end of life, when transfer to a hospital is distressing to both the resident and the family [56–58]. Evans et al. found that when physicians complied with the wishes of their patients, there was a 20% increase in the number of patients dying in the NH, rather than in the hospital [59].

In the ED, advance directives are useful to clinicians who often need to make treatment decisions without detailed knowledge of the patient’s history and wishes [10, 36, 49, 55, 57, 58]. Advance directives were available in 44% of the patients presenting at the ED, 64% had a do not resuscitate order, 60% had a health care proxy documented and 12% had a living will documented [52]. The presence of DNR orders in the NH reduced the odds of in-hospital death by 45–54% whereas do not hospitalise orders reduced the odds by 69–77% [57]. A study conducted by Nakashima et al. described that the absence of a do not hospitalise order was significantly associated with increased odds of hospital admission [58].

Although advance directives promote patient autonomy, problems with their use have been reported [6, 43, 51, 53, 55, 58, 60]. Pauls et al. described that only 4 to 8% of NH residents who were transferred to an ED had an advance directive available at ED transfer. Cohen et al. stated that the presence of do not hospitalise orders ranged from 2 to 8%. They also reported that NH staff felt that do not hospitalise orders are complex and open to interpretation depending on the situation [51, 60].

NH staff often feel pressure from relatives of a dying resident to seek or perform active treatment, including cardiopulmonary resuscitation and transfer to the ED. [32, 34, 50, 51] Relatives often had unrealistic expectations of the outcome of resuscitation. Staff recommended that relatives should be educated about end-of-life decisions [50]. Law suits against NHs by relatives of the residents result in an increased concern about liability issues [57]. NH staff and physicians were, therefore, more inclined to admit residents to a hospital at the end of life and in urgent situations, thus increasing the number of ED transfers and the risk of in-hospital death [4, 31, 57, 61, 62].

Several NH-related factors contribute to differences in hospital utilisation such as the quality of care delivered by nurses, education and number of available staff, physician’s availability at the NH, quality of the primary care, engagement in advance care planning and capability to deliver end-of-life care [13, 30, 34, 44, 49, 51, 61–63]. Gruneir et al. described that NHs who were located within 5 minutes of an ED, were of a larger size and had a historically high ED transfer rate, were associated with higher ED transfer rates [64].

The lack of availability of a GP was a potential explanation for the increase in ED transfers [4, 13, 34, 49, 51, 65]. Seven studies suggested that timely attendance by a GP allowed early medical assessment of an ill resident, early clinical diagnosis and initiation of treatment at the NH, reducing the number of ED transfers [6, 13, 27, 44, 63, 66, 67]. These GPs were usually not on-site; they only visited the NH through scheduled appointments and on an on-call basis [19, 40, 45, 51]. The number of pre-transfer contacts with GPs therefore was low and became even lower during out-of-hours [2, 13, 18, 27, 59, 68]. Briggs et al. found that in 40% of the ED transfers there was no prior GP visit. This number increased to 77% during the out-of-hours period [2]. Two observational studies in France showed that two-thirds of the ED admissions were referred by a treating physician [45, 46]. Other authors concluded that because of the low availability of general practitioners in some countries, NHs who were in need of acute health services were more likely to call the emergency medical services instead of a general practitioner [5, 7, 13, 28, 34].

## Discussion

### The lack of a uniform definition of “appropriateness”

The wide range of inappropriate ED transfers (reported between 4 and 55%) may be explained by the heterogeneity of facilities and their location, the subjectivity of the definition of appropriateness, and the extent to which facilities adopt measures that reduce inappropriate transfers [18, 31, 49, 69]. A universal definition would allow inappropriate transfers to be distinguished. Well-defined criteria would help NH staff, GPs, emergency medical services and EDs to make more appropriate decisions. The lack of consensus around the suitability of transfers and hospital admission suggests that concepts of “inappropriateness” are not shared by everyone who provides usual care for these patients [14, 15, 20, 69]. The term “inappropriate” should therefore be used with caution and each patient should be evaluated individually.

In the following sections we discuss recommendations for good clinical practice to allow health care systems to decrease inappropriate transfers to the ED.

### Respect the patient’s autonomy

NH residents generally do not want to be transferred to the ED, admitted, and die in a hospital [10, 56, 57]. It is important for NH staff and physicians to respect the wishes and concerns of the NH residents regarding advance care planning and end-of-life care planning. We, therefore, encourage the use of advance directives such as do not resuscitate, do not hospitalise and a health care proxy, as a way to convey the wishes of the NH residents to NH staff and physicians. A strong patient-physician relationship facilitates the discussion around delicate subjects as end-of-life care [10–12, 47, 62, 63]. When a health care proxy is used to make decisions for the NH resident, it is important to rule out any potential conflict of interest between the proxy and the NH resident.

### Provide acute treatment in the NH whenever possible

NH staff often believe that care in the ED is superior to care provided in the NH [31, 50, 62]. Acute medical conditions, however, can often be treated in the NH and high quality care in the NH avoids dying in a hospital. Especially for terminally ill patients, care in the NH is preferred over care in a hospital [13, 43]. Of residents who received the most integrated primary care only 5% died in a hospital, compared to 14–27% of those who received standard care [56]. But lack of organisation and poor quality of acute care delivered by NH staff are challenging [5, 31, 33, 38, 41, 51, 56, 57, 62, 70]. The quality of primary care services at NHs is influenced by multiple factors. These include the availability and the capability of nurses and physicians to manage chronic and acute conditions, end-of-life planning and fall prevention [13, 18, 33, 40, 41, 44, 70, 71]. For instance, instead of automatically triggering an ED transfer after a fall, careful monitoring, adequate documentation and an evaluation of fall risk factors can be provided in the NH [27, 31, 35, 40, 71].

### Provide sufficient and better educated nursing staff in the NH

Residents who need additional care put pressure on NH staff and resources. These residents, therefore, are more prone to be sent to the ED by NH staff to decrease their workload and costs for the NH [19, 57]. Surveys among NH staff revealed concerns about inadequate training and understaffing, particularly at night [34, 41, 51, 62]. When fewer skilled staff were available, monitoring of seriously ill patients became more difficult [18, 34, 51, 62]. More and better educated nursing staff such as registered nurses and (geriatric) nurse practitioners has shown to reduce the number of (inappropriate) hospitalisations and ED transfers through improved capacity to detect and to manage acutely ill residents on-site [5, 33, 34, 41, 44, 45, 49, 51, 56, 61–63, 65, 70, 72]. Providing better education in acute medical care allows NH staff to respond more adequately [6, 15, 20, 34, 41, 51, 62, 65, 72, 73].

A large European study performed in nine different countries and 300 hospitals showed that a higher nurse to patient ratio and better education decreased the likelihood of an inpatient dying within 30 days of admission. This suggests that higher education of nurses may reduce preventable hospital deaths and that cutting nurse staffing to save money might adversely affect patient outcomes [74].

### Improve the availability of GPs

By improving the availability of general practitioners during working hours as well as during out-of-hours, NH residents will have earlier access to appropriate medical care. This may increase appropriate referral decisions [9, 13, 15, 20, 41, 43, 51, 62, 67, 75]. Marshall et al. observed a reduction of 34% in overall transfers from NHs to EDs attributable to improved onsite primary care, with consistent physician and team engagement and improvements in continuity of care [13]. Additionally, by enhancing the continuity of care by GPs, the patient-physician relationship may improve. This in turn may facilitate more open discussion about delicate topics such as end-of-life care planning, which may decrease the number of transfers [62, 63, 66].

### Improve interprofessional collaboration and communication

Improving interprofessional collaboration between GPs, NH staff and EDs should be encouraged because it prevents inappropriate transfer of NH residents [13, 15, 20, 34, 50, 62, 67, 70]. Nursing staff will feel safer and more confident knowing why, when and how they can rely on a GP [13, 15, 19, 34, 37, 50, 51, 62]. To address future challenges in primary care, however, there is a need for more integrated interprofessional collaboration care models with sufficiently educated nurses [10, 15, 65, 76]. Moreover, because often a minority of nursing students choose the gerontology specialist option, gerontology content in basic nursing curricula should be increased [77]. A specialty level such as dedicated nurse practitioner will also help addressing the workload and general shortage of GPs for the treatment and care of NH residents [10, 51, 72, 78].

When primary care at the NHs is well organised and more resources are available, NH staff are more capable of managing acute situations [10, 13, 15, 19, 51, 56, 72]. For instance, by better prevention, immunisation and active treatment at the NH for common conditions, the amount of inappropriate ED transfers can be reduced [17, 47, 48, 70, 72, 79]. Fan et al. tested a program involving a responsive and dynamic team of ED-based nurses working in partnership and coordinating with NH staff. Implementing this “hospital in the NH”-program reduced the number of ED presentations by 17% and hospital admission rate by 37% [72]. Contrarily, the INTERACT program by Kane et al. did not result in a reduction in ED visit rates or hospitalisation, but it did reduce potentially avoidable hospitalisations by 15%. The INTERACT program included a set of tools that address the key factors leading to avoidable hospital admissions and ED visits among NH residents. INTERACT was based on 3 core principles: 1) recognition and management of acute conditions before they become severe enough to require hospitalisation; 2) providing communication, documentation, and decision support tools that allow for effective management in the NH without hospital admission when safe and feasible; 3) emphasising advance care planning, hospice and palliative care to encourage goals of care discussions and reduce hospitalisations in people with end-stage illness. The results of this study have several important implications for implementing quality improvement initiatives in NHs [61]. Implementing change will require financial resources which will be challenging certainly in view of an ageing population.

Telemedicine has been proposed as an innovative approach to improve access to care, thereby preventing ED visits. Telemedicine combines call center technology with formal or informal clinical decision systems to assess the health condition of patients and to provide advice about care [80–84]. Only a few studies have demonstrated the feasibility of telemedicine in the NH, in order to reduce ED utilisation. These studies report a 53% reduction in hospitalisations and a reduction of ED usage by 18 to 46%, without increasing GP visits or mortality [81, 83, 84].

### Limitations

Many articles in this review studied health care systems in the United Kingdom and in the United States. These countries have quite different healthcare and financial systems compared to continental Europe. Generalising their findings should therefore be done with caution. This underscores the need for more research on this subject in countries with different healthcare and financial systems. Nonetheless our main conclusions were supported by most studies, illustrating that the problem of inappropriate transfers from NHs to EDs and its causes is widespread. Most studies were observational and therefore the strength of the evidence was weak. Due to the subjective nature of the topic and the overall quality of the articles, our conclusions and recommendations have a low degree of certainty.

## Conclusion

The most common reasons for transfer of NH residents to EDs are trauma after falling, altered mental status and infection. The evidence in this review shows that 4 to 55% of transfers of NH residents to EDs were inappropriate. The definition of appropriateness is not uniform across studies and needs further investigation. To avoid inappropriate transfer to EDs, we recommend to respect the patient’s autonomy, to provide sufficient nursing staff and to invest in their education, to increase the role of GPs in the care of NH residents both in standard and in acute situations, and to promote interprofessional communication and collaboration between GPs, NH staff and EDs.

## Additional files

Additional file 1: **Appendix 1.** JBI Critical Appraisal Checklist for Narrative Expert opinion & text. **Appendix 2.** STROBE checklist. **Appendix 3.** COREQ checklist. **Appendix 4.** AMSTAR checklist. **Appendix 5.** JBI Critical Appraisal Checklist for Quasi-Experimental Studies. **Appendix 6.** CONSORT Checklist. The checklists were used to assess the studies. (DOCX 71 kb)

Additional file 2: **Table S3.** Characteristics of included studies concerning “Appropriateness”. These are the characteristics of the included studies. (DOCX 16 kb)

## Abbreviations

DNR: Do Not Resuscitate
ED: Emergency Department AD Advance Directive
GP: General Practitioner
NH: Nursing Home
