# Supplementary material for: Appropriateness of transferring nursing home residents to emergency departments: a systematic review
Source: BMC Geriatr. 2019 Jan 21;19:17. doi: 10.1186/s12877-019-1028-z (PMC6341611; doi:10.1186/s12877-019-1028-z)
Supplement: Supplementary file 1 — Appendix 1. JBI Critical Appraisal Checklist for Narrative Expert opinion & text. Appendix 2. STROBE checklist. Appendix 3. COREQ checklist. Appendix 4. AMSTAR checklist. Appendix 5. JBI Critical Appraisal Checklist for Quasi-Experimental Studies. Appendix 6. CONSORT Checklist. The checklists were used to assess the studies. (DOCX 71 kb) [file 12877_2019_1028_MOESM1_ESM.docx]

**Appendix**

**Appendix 1**

| **JBI Critical Appraisal Checklist for Narrative Expert opinion & text** | | | | | | |
| --- | --- | --- | --- | --- | --- | --- |
| Item No | Gruneir, 2013 | McCloskey, 2007 | Wofford, 1993 | Kahn, 2016 | Ouslander, 2015 | Bodenheimer, 2016 |
| 1 | Yes | Yes | Yes | Yes | Yes | Yes |
| 2 | No | Yes | Yes | Yes | Yes | Yes |
| 3 | No | Yes | Yes | Yes | Yes | Yes |
| 4 | Yes | Yes | Yes | Yes | Yes | Yes |
| 5 | Yes | Yes | Yes | Yes | Yes | Yes |
| 6 | Yes | Yes | Yes | Yes | Yes | Yes |
| 7 | Yes | Yes | Yes | Yes | Yes | Yes |

**Appendix 2**

| **STROBE checklist (part 1)** | | | | | | | | | |
| --- | --- | --- | --- | --- | --- | --- | --- | --- | --- |
| Item No | Amador, 2014 | Arendts, 2012 | Briggs, 2013 | Brownell, 2014 | Bynum, 2011 | Caffrey, 2010 | Carter, 2009 | Codde, 2010 | Evans, 2011 |
| 1 a) | yes | yes | yes | yes | yes | no | yes | yes | yes |
| 1 b) | yes | yes | yes | no | yes | no | yes | yes | yes |
| 2 | yes | yes | yes | yes | yes | no | yes | yes | yes |
| 3 | yes | yes | yes | yes | yes | no | yes | yes | no |
| 4 | yes | yes | yes | yes | yes | yes | yes | yes | yes |
| 5 | yes | yes | yes | no | yes | yes | yes | yes | yes |
| 6 a) | yes | yes | yes | yes | yes | yes | yes | yes | no |
| 6 b) | no | no | no | no | no | no | no | no | no |
| 7 | yes | yes | yes | no | no | yes | no | no | yes |
| 8 | yes | yes | no | no | yes | yes | yes | no | no |
| 9 | no | yes | no | no | yes | no | yes | no | no |
| 10 | yes | yes | yes | yes | yes | yes | yes | yes | no |
| 11 | no | yes | no | no | yes | no | no | no | no |
| 12 a) | yes | yes | no | yes | yes | yes | no | no | no |
| 12 b) | no | yes | yes | no | no | no | no | no | no |
| 12 c) | yes | no | no | no | no | no | no | no | no |
| 12 d) | yes | no | no | no | no | no | no | no | no |
| 12 e) | no | no | no | no | no | no | no | no | no |
| 13 a) | yes | yes | yes | no | yes | no | yes | yes | yes |
| 13 b) | yes | no | no | yes | no | no | yes | no | no |
| 13 c) | no | yes | no | no | no | no | no | no | no |
| 14 a) | yes | yes | yes | yes | yes | yes | yes | yes | yes |
| 14 b) | no | no | no | no | no | no | no | yes | no |
| 14 c) | yes | no | yes | yes | no | no | no | no | yes |
| 15 | yes | yes | yes | yes | yes | yes | yes | yes | yes |
| 16 a) | yes | yes | yes | no | no | no | no | no | no |
| 16 b) | no | no | no | no | no | no | no | no | no |
| 16 c) | no | no | no | no | no | no | no | no | no |
| 17 | yes | no | yes | yes | yes | yes | yes | yes | yes |
| 18 | yes | yes | yes | yes | yes | yes | yes | yes | yes |
| 19 | yes | no | yes | no | no | yes | yes | yes | no |
| 20 | yes | yes | yes | yes | yes | no | yes | yes | yes |
| 21 | yes | no | yes | no | no | no | no | yes | no |
| 22 | no | yes | no | no | no | no | no | yes | no |

| **STROBE checklist (part 2)** | | | | | | | | | | |
| --- | --- | --- | --- | --- | --- | --- | --- | --- | --- | --- |
| Item No | Gruneir, 2010 | Hutt, 2002 | Kirsebom, 2014 | Lahn, 2001 | McGregor, 2014 | Mitchell, 2010 | Quinn, 2011 | Saliba, 2000 | Temkin-Greener, 2013 | Aiken, 2014 |
| 1 a) | yes | yes | yes | yes | yes | yes | yes | yes | yes | yes |
| 1 b) | yes | yes | yes | yes | yes | yes | yes | yes | yes | yes |
| 2 | yes | yes | yes | yes | yes | yes | yes | yes | yes | yes |
| 3 | yes | yes | yes | yes | yes | yes | yes | yes | yes | yes |
| 4 | yes | yes | yes | yes | yes | yes | yes | yes | yes | yes |
| 5 | yes | yes | yes | yes | yes | yes | no | yes | yes | yes |
| 6 a) | yes | yes | yes | yes | yes | yes | yes | yes | yes | yes |
| 6 b) | no | no | no | no | yes | no | no | no | yes | no |
| 7 | yes | yes | no | yes | no | no | no | no | yes | yes |
| 8 | yes | yes | no | no | no | no | no | no | yes | yes |
| 9 | yes | no | no | yes | yes | no | yes | yes | yes | no |
| 10 | yes | yes | yes | yes | yes | yes | yes | yes | yes | yes |
| 11 | no | no | no | no | no | no | no | no | yes | no |
| 12 a) | yes | yes | yes | yes | yes | no | yes | no | yes | no |
| 12 b) | no | yes | yes | no | no | no | no | no | yes | no |
| 12 c) | no | no | no | no | no | no | no | no | no | no |
| 12 d) | no | no | no | no | no | no | no | no | no | no |
| 12 e) | no | no | no | yes | no | no | no | yes | no | no |
| 13 a) | yes | yes | yes | yes | yes | yes | yes | yes | yes | yes |
| 13 b) | yes | no | no | no | yes | no | no | yes | no | no |
| 13 c) | no | no | no | no | no | no | no | yes | no | no |
| 14 a) | yes | yes | yes | yes | yes | yes | yes | yes | yes | yes |
| 14 b) | no | no | no | no | no | no | no | yes | no | yes |
| 14 c) | yes | no | yes | no | yes | yes | no | yes | yes | yes |
| 15 | yes | yes | yes | no | yes | yes | yes | yes | yes | yes |
| 16 a) | no | yes | no | yes | no | no | yes | yes | yes | yes |
| 16 b) | no | no | no | no | no | no | no | no | no | no |
| 16 c) | no | no | no | no | no | no | no | no | no | no |
| 17 | yes | yes | yes | yes | yes | no | yes | yes | yes | yes |
| 18 | yes | yes | yes | yes | yes | yes | yes | yes | yes | yes |
| 19 | yes | yes | yes | yes | yes | yes | yes | yes | yes | yes |
| 20 | yes | yes | yes | yes | yes | yes | yes | yes | yes | yes |
| 21 | no | no | no | yes | yes | yes | yes | no | no | yes |
| 22 | no | no | yes | no | yes | yes | yes | yes | yes | yes |

| **STROBE checklist (part 3)** | | | | | | | | | | | | |
| --- | --- | --- | --- | --- | --- | --- | --- | --- | --- | --- | --- | --- |
| Item No | Burke, 2015 | Carron, 2015 | Morphet, 2015 | Lee, 2003 | Carron, 2017 | Fan, 2015 | Gruneir, 2016 | Nakaskima, 2013 | Ouslander, 2016 | Faul, 2016 | Wang, 2011 | O'connell, 2013 |
| 1 a) | yes | yes | yes | yes | yes | yes | yes | yes | yes | yes | yes | no |
| 1 b) | yes | yes | yes | yes | yes | yes | yes | yes | yes | yes | yes | yes |
| 2 | yes | yes | yes | yes | yes | yes | yes | yes | yes | yes | yes | yes |
| 3 | yes | yes | yes | yes | yes | yes | yes | yes | yes | yes | yes | yes |
| 4 | yes | yes | yes | yes | yes | no | yes | yes | yes | yes | yes | yes |
| 5 | yes | yes | yes | yes | yes | yes | yes | yes | yes | yes | yes | yes |
| 6 a) | yes | yes | yes | yes | yes | yes | yes | no | yes | yes | yes | yes |
| 6 b) | no | no | no | no | no | no | no | no | yes | yes | yes | no |
| 7 | no | no | no | no | yes | yes | yes | yes | yes | yes | yes | yes |
| 8 | no | no | no | yes | no | no | yes | yes | yes | yes | yes | no |
| 9 | no | no | no | no | no | no | no | no | yes | no | no | no |
| 10 | yes | yes | yes | yes | yes | yes | yes | no | yes | yes | yes | yes |
| 11 | yes | yes | yes | yes | no | no | no | yes | yes | yes | yes | no |
| 12 a) | no | no | no | yes | no | no | yes | yes | no | no | no | no |
| 12 b) | no | no | no | yes | no | no | no | no | no | no | no | no |
| 12 c) | no | no | yes | yes | no | no | no | no | yes | no | no | no |
| 12 d) | no | no | no | no | no | no | no | no | no | no | no | no |
| 12 e) | no | no | no | no | no | no | no | yes | no | no | no | no |
| 13 a) | no | no | yes | yes | yes | no | no | yes | yes | yes | yes | no |
| 13 b) | no | no | no | no | no | no | no | no | no | no | no | no |
| 13 c) | no | no | yes | no | no | no | no | no | no | no | no | no |
| 14 a) | no | no | no | yes | yes | yes | yes | yes | yes | yes | yes | yes |
| 14 b) | no | no | no | no | no | no | no | no | yes | no | no | no |
| 14 c) | no | yes | no | no | yes | yes | yes | no | yes | yes | yes | yes |
| 15 | yes | yes | yes | yes | yes | yes | yes | yes | yes | yes | yes | yes |
| 16 a) | no | no | no | yes | yes | yes | yes | yes | yes | yes | yes | yes |
| 16 b) | no | no | no | yes | no | no | no | no | no | no | no | no |
| 16 c) | no | no | no | no | no | no | no | no | no | no | no | no |
| 17 | no | no | no | no | yes | yes | yes | no | yes | yes | yes | yes |
| 18 | yes | yes | yes | yes | yes | yes | yes | yes | yes | yes | yes | yes |
| 19 | yes | yes | yes | yes | yes | yes | yes | yes | yes | yes | yes | yes |
| 20 | yes | yes | yes | yes | yes | yes | yes | yes | yes | yes | yes | yes |
| 21 | yes | yes | yes | yes | yes | yes | yes | yes | yes | yes | yes | yes |
| 22 | yes | yes | no | no | no | no | no | no | no | no | no | no |

| **STROBE checklist (part 4)** | | | | | | | | | | | | | |
| --- | --- | --- | --- | --- | --- | --- | --- | --- | --- | --- | --- | --- | --- |
| Item No | Menand, 2015 | Marshall, 2016 | Manckoundia, 2016 | Jensen, 2009 | Haber, 2017 | Girio-Fragkoulakis, 2011 | Carter, 2006 | Bowman, 2001 | Witt, 2013 | Bollig, 2008 | Caplan, 2006 | Kada, 2011 | McQuown, 2017 |
| 1 a) | yes | no | yes | yes | yes | yes | yes | no | yes | yes | yes | yes | yes |
| 1 b) | yes | yes | yes | yes | yes | yes | yes | no | no | yes | yes | yes | yes |
| 2 | yes | yes | yes | yes | yes | yes | yes | yes | yes | yes | yes | yes | yes |
| 3 | yes | yes | yes | yes | yes | yes | yes | yes | no | yes | yes | yes | yes |
| 4 | yes | yes | yes | yes | yes | yes | yes | yes | no | yes | yes | yes | yes |
| 5 | yes | yes | yes | yes | yes | yes | yes | yes | yes | yes | yes | yes | yes |
| 6 a) | yes | yes | yes | yes | yes | no | yes | yes | yes | yes | yes | yes | yes |
| 6 b) | yes | no | yes | yes | no | no | no | no | no | no | no | no | no |
| 7 | yes | yes | yes | yes | yes | yes | yes | no | no | yes | yes | yes | yes |
| 8 | yes | yes | yes | yes | yes | yes | yes | no | no | no | yes | yes | yes |
| 9 | no | no | no | no | no | yes | yes | no | no | no | no | no | no |
| 10 | yes | yes | yes | yes | yes | no | yes | yes | yes | yes | yes | yes | yes |
| 11 | yes | yes | no | no | no | no | no | no | no | no | no | no | no |
| 12 a) | no | yes | no | no | yes | no | no | no | no | no | no | no | no |
| 12 b) | no | yes | no | yes | no | no | no | no | no | no | no | no | no |
| 12 c) | yes | yes | yes | no | yes | no | no | no | no | no | no | no | no |
| 12 d) | no | yes | no | no | yes | no | no | no | no | no | no | no | no |
| 12 e) | no | no | no | no | no | yes | no | no | no | no | no | no | no |
| 13 a) | yes | yes | yes | no | yes | yes | yes | no | no | no | no | no | no |
| 13 b) | no | no | yes | no | yes | no | no | no | no | no | no | no | no |
| 13 c) | yes | no | no | no | no | yes | no | no | no | no | no | no | no |
| 14 a) | yes | yes | no | yes | yes | no | yes | yes | yes | yes | yes | yes | yes |
| 14 b) | yes | yes | no | no | no | no | no | no | no | no | no | no | no |
| 14 c) | yes | yes | yes | yes | yes | yes | yes | yes | yes | yes | yes | yes | yes |
| 15 | yes | yes | yes | yes | yes | no | yes | yes | yes | yes | yes | yes | yes |
| 16 a) | yes | yes | yes | yes | yes | no | yes | yes | yes | yes | yes | yes | yes |
| 16 b) | no | yes | yes | no | no | no | no | no | no | no | yes | no | no |
| 16 c) | no | yes | yes | no | no | yes | no | no | no | no | no | no | no |
| 17 | yes | yes | yes | yes | yes | yes | yes | yes | no | yes | yes | yes | yes |
| 18 | yes | yes | yes | yes | yes | yes | yes | yes | yes | yes | yes | yes | yes |
| 19 | yes | yes | yes | yes | yes | yes | yes | yes | yes | yes | yes | yes | yes |
| 20 | yes | yes | yes | yes | yes | no | yes | yes | yes | yes | yes | yes | yes |
| 21 | yes | yes | yes | yes | yes | yes | yes | yes | yes | yes | yes | yes | yes |
| 22 | no | yes | no | no | yes | no | no | no | no | no | yes | yes | no |

**Appendix 3**

| **COREQ checklist** | | | | | | | | | | |
| --- | --- | --- | --- | --- | --- | --- | --- | --- | --- | --- |
| Item No | Arendts, 2010 | Crilly, 2012 | Pauls, 2001 | Arendts, 2015 | Shah, 2013 | Amaduro, 2018 | Deschodt, 2010 | Stokoe, 2015 | Cohen, 2017 | Harisson, 2016 |
| 1 | N/A | p.12 | p.2 | p. 1 | p.2/3 | p 2 | N/A | p.1 | p.4 | p.1 |
| 2 | p.1 | p.1 | p.1 | p. 1 | p.1 | p.1 | p.1 | p.1 | p.1 | p.1 |
| 3 | p.1 | p.12 | N/A | p. 1 | p.1 | p.1 | p.1 | p.1 | p.1 | p.1 |
| 4 | p.1 | p.1 | N/A | p. 1 | p.1 | p.1/2 | p.1 | p.1 | p.1 | p.1 |
| 5 | p.1/2 | N/A | N/A | N/A | N/A | p.1/2 | N/A | p.2 | p.4 | N/A |
| 6 | p.1/2 | N/A | N/A | p. 2 | N/A | p.2 | N/A | p.2 | p.3 | N/A |
| 7 | p.1/2 | N/A | N/A | N/A | N/A | p.2 | N/A | N/A | N/A | N/A |
| 8 | N/A | N/A | N/A | N/A | N/A | N/A | N/A | N/A | p.4 | N/A |
| 9 | p.1 | p.1/2 | p.1/2 | N/A | p.1/2 | N/A | p.2/3 | N/A | p.4 | p.2 |
| 10 | p.1/2 | p.2/3 | p.2 | p.2 | p.2/3 | p.2 | p.3 | p.2 | p.3 | p.2 |
| 11 | p.1/2 | p.3 | p.2 | p.2 | p.2 | p.2 | N/A | p.2 | p.3 | p.2 |
| 12 | N/A | p.3/4 | p.2 | p.2 | p.1 | p.2 | p.4 | p.2 | p.3/4 | p.2 |
| 13 | N/A | N/A | N/A | N/A | N/A | N/A | N/A | N/A | p.3 | p.2 |
| 14 | N/A | p.3 | N/A | p. 1 | N/A | p.2 | N/A | p.2 | p.3 | p.2 |
| 15 | p.2 | p.3 | p.2 | N/A | p.2 | p.2 | N/A | p.2 | p.4 | N/A |
| 16 | p.1/2 | p.3/4 | p.3 | p.2 | p.3 | p.2 | p.3 | p.2 | p.3 | p.2 |
| 17 | N/A | p.3 | p.2 | N/A | N/A | N/A | N/A | p.2 | N/A | p.2 |
| 18 | p.1/2 | N/A | N/A | N/A | N/A | N/A | p.3/4 | N/A | N/A | N/A |
| 19 | p.2 | p.3 | p.2 | p.2 | p.2 | p.2/3 | N/A | p.2 | N/A | N/A |
| 20 | p.2 | N/A | N/A | p.2 | p.2 | p.2 | N/A | p.2 | p.4 | N/A |
| 21 | N/A | p.3 | N/A | N/A | N/A | p.2 | N/A | p.2 | p.4 | N/A |
| 22 | N/A | N/A | N/A | p.2 | p.1/2 | N/A | N/A | N/A | p.3 | N/A |
| 23 | p.2 | N/A | N/A | N/A | N/A | p.3 | N/A | p.2 | N/A | N/A |
| 24 | N/A | p.12 | p.2 | p.2 | p.3 | p.3 | N/A | p.2 | p.4 | p.2 |
| 25 | N/A | p.3 | N/A | p.2 | N/A | N/A | N/A | N/A | p.4 | N/A |
| 26 | p.1/2 | p.3 | p.2 | p.2 | p.2 | p.3 | p.1/2 | p.2/3 | p.4 | p.2 |
| 27 | N/A | N/A | p.2 | p.2 | N/A | N/A | p.3 | N/A | p.4 | p.2 |
| 28 | p.2/3 | N/A | N/A | N/A | N/A | p.3 | N/A | p.2 | N/A | N/A |
| 29 | p.2/3 | p.6/7/8 | p.5 | p.2/3/4/5 | N/A | p.3/4/5/6 | N/A | p.3/4 | p.5/6 | p.4 |
| 30 | p.2/3 | p.6/7/8/9 | p. 3/4/5 | p.5 | p.4/5 | p.3/4/5/6 | p.4/5/6 | p.3/4 | p.5/6 | p.3/4/5 |
| 31 | p.2/3 | p.6/7/8/10 | p. 3/4/5 | p.2/3/4/5 | p.3/4/5 | p.3/4/5/6 | p.1/2 | p.3 | p.5 | p.4 |
| 32 | p.2/3/4 | N/A | N/A | N/A | N/A | N/A | p.6/7 | p.3/4 | N/A | N/A |

**Appendix 4**

| **AMSTAR checklist** | | | | | | | | | |
| --- | --- | --- | --- | --- | --- | --- | --- | --- | --- |
| Item No | Dwyer, 2014 | Graverholt, 2014 | Kessler, 2013 | Renom-guiteras, 2014 | Trahan, 2016 | Dwyer, 2015 | Matthys, 2017 | Arendts, 2013 | Carrasqueiro, 2011 |
| 1 | Yes | Yes | Yes | Yes | Yes | Yes | Yes | Yes | Yes |
| 2 | No | Yes | No | Yes | Yes | No | Yes | No | Yes |
| 3 | Yes | Yes | Yes | Yes | Yes | Yes | Yes | Yes | No |
| 4 | No | No | No | No | Yes | No | No | No | No |
| 5 | No | Yes | No | Yes | No | Yes | No | No | No |
| 6 | No | Yes | No | Yes | Yes | Yes | Yes | No | No |
| 7 | No | Yes | No | No | Yes | Yes | Yes | No | No |
| 8 | No | Yes | No | No | Yes | No | Yes | No | No |
| 9 | No | Yes | No | No | No | No | Yes | No | No |
| 10 | No | No | No | No | No | No | No | No | No |
| 11 | No | No | No | No | No | No | No | No | No |
| Total score | 2 | 8 | 2 | 4 | 7 | 5 | 7 | 2 | 2 |

**Appendix 5**

| **JBI Critical Appraisal Checklist for Quasi-Experimental Studies** | | | | | | |  |
| --- | --- | --- | --- | --- | --- | --- | --- |
| Item No | Codde, 2010 | Pain, 2014 | Fan, 2016 | Hullick, 2016 | Mierdel, 2015 | Shah, 2015 | Shah, 2016 |
| 1 | Yes | Yes | Yes | Yes | Yes | Yes | Yes |
| 2 | Yes | Yes | Yes | Yes | Yes | Yes | Yes |
| 3 | Yes | No | Yes | Yes | Yes | Yes | Yes |
| 4 | No | No | Yes | Yes | No | Yes | Yes |
| 5 | No | Yes | Yes | Yes | yes | No | No |
| 6 | No | No | Yes | Yes | yes | Yes | Yes |
| 7 | Yes | Yes | Yes | Yes | yes | Yes | Yes |
| 8 | Yes | Yes | Yes | Yes | yes | Yes | Yes |
| 9 | Yes | No | Yes | Yes | yes | Yes | Yes |
| Total score | 6 | 5 | 9 | 9 | 8 | 8 | 8 |

**Appendix 6**

| **CONSORT checklist** | |
| --- | --- |
| Item No | Kane, 2017 |
| 1a | p.1 |
| 1b | p.1 |
| 2a | p.2 |
| 2b | p.2 |
| 3a | p.2 |
| 3b | p.3 |
| 4a | p.3 |
| 4b | p.3 |
| 5 | p.2/3 |
| 6a | p.3 |
| 6b | p.3 |
| 7a | N/A |
| 7b | N/A |
| 8a | p.3 |
| 8b | p.3 |
| 9 | p.3 |
| 10 | p.3 |
| 11a | N/A |
| 11b | N/A |
| 12a | p.3 |
| 12b | p.3 |
| 13a | p.4 |
| 13b | p.4 |
| 14a | p.3 |
| 14b | p.3 |
| 15 | p.4 |
| 16 | p.4 |
| 17a | p.4/5/6 |
| 17b | p.4/5/6 |
| 18 | p.4/5/6 |
| 19 | N/A |
| 20 | p.7 |
| 21 | N/A |
| 22 | p.6/7 |
| 23 | p.1 |
| 24 | p.1 |
| 25 | p.8 |
