# Supplementary material for: Appropriateness of transferring nursing home residents to emergency departments: a systematic review
Source: BMC Geriatr. 2019 Jan 21;19:17. doi: 10.1186/s12877-019-1028-z (PMC6341611; doi:10.1186/s12877-019-1028-z)
Supplement: Supplementary file 2 — Table S3. Characteristics of included studies concerning “Appropriateness”. These are the characteristics of the included studies. (DOCX 16 kb) [file 12877_2019_1028_MOESM2_ESM.docx]

Table S3: Characteristics of included studies concerning “Appropriateness”

| **Characteristics of Included Studies** | | | | | | |
| --- | --- | --- | --- | --- | --- | --- |
| **Author** | **Year** | **Country** | **Definition of “Appropriateness”** | **Methodology** | **Sample** | **Outcome: number of inappropriate visits/transfers – hospitalization rate** |
| Briggs et al. | 2013 | Ireland | - A ‘potentially preventable’ attendance: one that may have been avoided if optimal management of an existing condition was available in the NH at an earlier stage.  - A ‘low acuity’ visit: one rated as standard or non-urgent and not requiring in-patient management, resulting in direct discharge from ED. | Prospective chart review  Single site | N= 155 | - Potentially preventable: 55%  - Low acuity: 23%  - Hospitalization rate: 70% |
| Burke et al. | 2015 | USA | ‘Potentially preventable ED visits’: patients who do not undergo diagnostic testing and are discharged from the ED on the same day. | Retrospective analysis | N= 3857 | - Potentially preventable: 18.9%  - Hospitalisation rate: 46.5% |
| Caffrey et al. | 2010 | USA | Conditions associated with potentially preventable ED visits and hospitalisations. | Retrospective analysis | N= 123,600 | - Potentially preventable: 40% |
| Carter et al. | 2009 | United Kingdom | Appropriateness was defined by three experienced GP principals as to whether patient care could have been delivered differently without the ED. | Prospective single centre study | N= 107 | - Between 8 to 40% could have been managed appropriately without the ED. |
| Codde et al. | 2010 | Australia | - Potentially avoidable ED transfers are defined by a list of symptoms and diagnosis in which hospitalisation is generally considered unnecessary.  - Patients with high triage categories, suspected fractures, requiring technical investigations, abnormal parameters , neurological symptoms, the need for i.v. medication or fluid and/or requiring hospitalisation, were considered “appropriate” transfers. | Chart review  Single tertiary hospital ED | N= 235 | - Potentially preventable: 31% |
| Gruneir et al. | 2010 | Canada | - Cfr. Briggs et al. 2013 | Retrospective chart review | N= 64, 589 | - Potentially preventable: 24.6%  - Low acuity: 10.6%  - Hospitalisation: 43.8% |
| Jensen et al. | 2009 | Canada | ‘Appropriateness’ was defined as a balance of issues concerning availability of diagnostic and treatment resources, physician and nursing availability and expertise, advanced directives, respect for patient or family wishes, availability of background medical information, and premorbid health status. | Retrospective analysis | N= 606 | - Not appropriate: 32.7%.  - Hospitalisation rate: 62.5% |
| Kirsebom et al. | 2014 | Sweden | “Avoidable” was defined by the Swedish Association of Local Authorities and Regions (SALAR), indicating some acute and chronic conditions can be treated with favourable results in primary care, if given adequate and timely treatment. | Retrospective  descriptive design | N= 594 | - Avoidable hospital admissions: 16%  - Hospitalisation rate: 63% |
| Manckoundia et al. | 2012 | France | - The admission is considered appropriate if it meets one of the Appropriateness Evaluation Protocol (French version, AEPf) criteria.  - In doubt about the appropriateness of admission an expert committee ruled on the appropriateness of the admission to the ED. | Prospective multicentre study | N= 1000 | - Inappropriate hospital admissions: 18.1% |
| Menand et al. | 2015 | France | - Cfr. Manckoundia et al. 2012 | Prospective, cross-sectional, multicentre study | N= 1577 | - Inappropriate hospital admissions: 7.7% |
| Morphet et al. | 2015 | Australia | - Cfr. Codde et al. 2010 | Retrospective review of ED records  Two EDs | N= 408 | - Presenting complaint met the avoidability criteria: 35.3%  - Hospitalisation rate: 42.7% |
| Ouslander et al. | 2016 | USA | - Trained staff rated whether a transfer was potentially preventable or nonpreventable, using the INTERACT QI tool (a structured, retrospective root cause analysis of hospital transfers). | Retrospective analysis | N= 4527 | - Potentially preventable hospital transfers: 23% |
| Saliba et al. | 2000 | USA | - ‘Avoidable transfers’: when a patient’s health condition could have been safely managed inside the NH.  - Structured Implicit Review (SIR) to assess factors contributing to avoidability. | Retrospective analysis | N= 128 | - Inappropriate ED transfers: 36%  - Inappropriate hospital admissions: 40%  - Hospitalisation rate: 83% |
